# Effect of Shexiang Tongxin dropping pill on stable coronary artery disease patients with normal fractional flow reserve and coronary microvascular disease: A study protocol

**DOI:** 10.1097/MD.0000000000022126

**Published:** 2020-09-18

**Authors:** Yanli Lu, Xiaopeng Chu, Jiefang Zhang, Yanbo Zhao, Chongying Jin, Junhui Zhu, Guosheng Fu, Fuyu Qiu

**Affiliations:** Department of Cardiology, Key Laboratory of Cardiovascular Intervention and Regenerative Medicine of Zhejiang Province, Sir Run Run Shaw Hospital, Zhejiang University School of Medicine, Hangzhou, Zhejiang, China.

**Keywords:** coronary microvascular disease, Shexiang Tongxin dropping pill, stable coronary artery disease, study protocol

## Abstract

**Introduction:**

Coronary microvascular disease (CMVD) can affect the structure, function, and metabolism of the heart, and has an important impact on the occurrence, development and prognosis of coronary artery disease (CAD). Shexiang Tongxin dropping pill (STDP) can dilate blood vessels, alleviate inflammation, reduce endothelial damage, and improve coronary microvascular function in mice with myocardial infarction. This study aims to assess the impact of STDP on stable coronary artery disease (SCAD) patients with normal FFR and CMVD.

**Methods and analysis:**

This is a single-center, prospective randomized trial that will enroll 64 SCAD patients, CAD with normal FFR and CMVD. Patients will be randomly divided into study group and control group in a 1:1 fashion. On the basis of conventional drug treatment, the former will receive STDP while the latter will not. The follow-up period of the subjects is 12 months, and clinical follow-up will be conducted before discharge, 30 days, 3 months, 6 months, and 12 months after procedure to complete the detection of relevant indicators. The primary endpoint is the change of index of microcirculatory resistance (ΔIMR) at 12-month follow-up.

**Discussion:**

The present study will be the first randomized control study to evaluate the efficacy and safety of STDP on SCAD patients, CAD with normal FFR and CMVD, which will provide a broader idea and more experimental basis for improving the treatment of CMVD.

**Trial registration:**

This is a protocol for the randomized clinical trial which has been registered in the Chinese clinical Trial Registry with an identifier: ChiCTR2000032429.

## 1 Introduction

Coronary artery disease (CAD) is a common atherosclerotic cardiovascular disease that seriously harms human health. Among them, in some patients with CAD, the symptoms are caused by localized narrowing of the epicardial coronary arteries, which can be alleviated by drug therapy and revascularization if necessary. However, there is still a considerable part of patients with CAD, whose fractional flow reserve (FFR) test is normal but evidence of myocardial ischemia still exist, which may be associated with coronary microvascular disease (CMVD).

CMVD refers to the clinical syndrome of objective evidence of exertional angina or myocardial ischemia caused by abnormal structure and/or function of coronary prearterioles and arterioles under the action of various pathogenic factors.^[1]^ Ample evidence has revealed that microvascular structure changes or remodeling have been related to CMVD. Meanwhile, endothelial dysfunction in resistance coronary vessels is an extremely important contributor to CMVD.^[2]^ The WISE study included female patients with normal coronary angiography and reduced coronary flow reserve (CFR) and found that the incidence of cardiac death, stroke, and heart failure increased by 24% after 5 years.^[3]^ Spoladore found that changes in coronary microvascular blood flow, especially coronary endothelial dysfunction was the main cause of impaired cardiac function.^[4]^ Therefore, timely diagnosis and treatment of CMVD will effectively improve the symptoms and prognosis of the patients.

After study for over 20 years, the invasive and non-invasive assessment of coronary microvascular function has been established. Because of lack of regional perfusion abnormalities typically seen in obstructive CAD, traditional non-invasive ischemia tests may be normal in patients with CMVD.^[5]^ It is thought that cardiac magnetic resonance (CMR) imaging holds most promise as the first choice for noninvasive imaging.^[5]^ Invasive tests including CFR and index of microcirculatory resistance (IMR) are the reference standard for the diagnosis of CMVD. IMR is an index to evaluate the function of distal microvessel in patients with coronary artery stenosis, characterized by high specificity and good repeatability.^[6]^ Value of IMR ≥ 25 units is indicative of abnormal microcirulatory function.^[7]^

The treatment of CMVD has so far been empirical based on traditional therapies for CAD and the curative effect is not evident. The application of traditional Chinese medicine has opened up new ideas for us. Shexiang Tongxin dropping pill (STDP) is a traditional China Food and Drug Administration (CFDA)-approved prescription medicine (approval no. Z20080018), based on the ancient prescriptions “Zhi Bao Dan” and “Liu Shen Pills,” which consists of a combination of seven valuable drugs such as artificial musk, secretio bufonis, total saponins of ginseng, salvia miltiorrhiza, artificial bezoar, bear gall, borneol, etc. Studies have shown that STDP can increase the expression of endogenous nitric oxide synthase (eNOS) gene, increase the content of NO in plasma, and thus play a role in vasodilating, inhibiting platelet aggregation, scavenging oxygen free radicals so as to improve microcirculation dysfunction.

However, there is no clinical study to observe and confirm whether STDP can improve the microvascular function of patients with CAD. Thus, a prospective randomized controlled trial will be designed to address the issue. We will test the hypothesis that STDP can significantly improve the coronary microvascular function in stable CAD (SCAD) patients with normal FFR and CMVD. In this article, we describe the study design discuss the rationale for the specific approaches employed.

## 2 Methods and analysis

### 2.1 Study design

**Figure 1:**
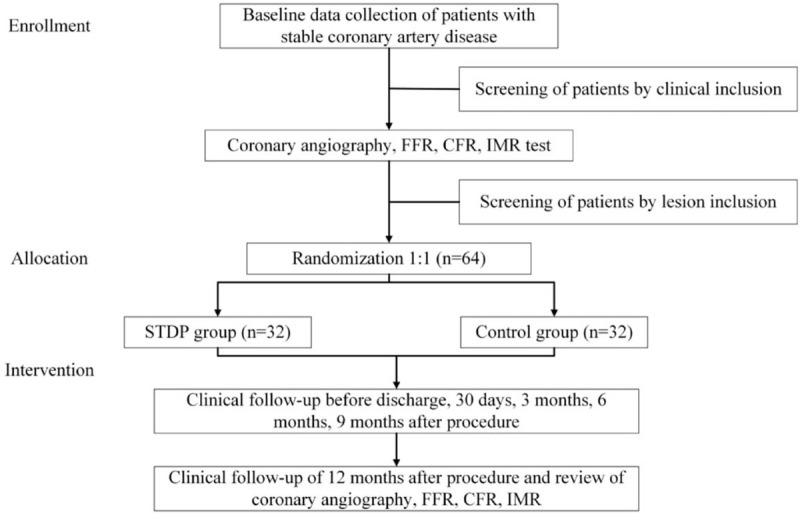
Study flow chart.

This is a single-center, prospective randomized trial that will enroll 64 SCAD patients with normal FFR and CMVD. The protocol has been approved by the Ethics Committee of Sir Run Run Show Hospital Affiliated to Zhejiang University School of Medicine (No.20200423–36). Participants who meet the eligibility criteria will be enrolled and randomly allocated to study group and control group in a 1:1 fashion. On the basis of conventional drug treatment, the former will receive STDP while the latter will not. The follow-up period of the subjects was 12 months, and clinical follow-up will be conducted before discharge, 30 days, 3 months, 6 months, and 12 months after procedure to complete the detection of relevant indicators. The study flow chart is shown in Figure 1.

Corresponding parameters shown in Table 1 including laboratory tests and other indicators will be recorded or assessed at each follow-up by the responsible physician.

**Table 1:**
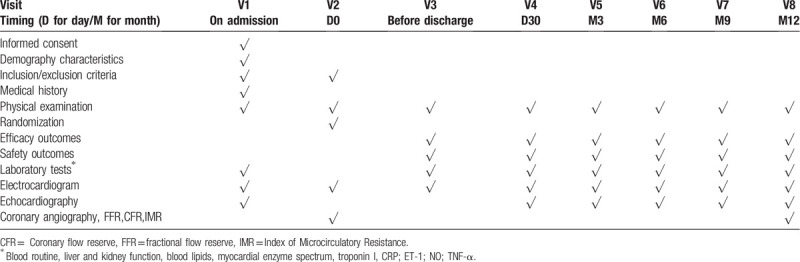
Planned visits and parameters.

### 2.2 Hypothesis

This study hypothesizes that for SCAD patients with normal FFR and CMVD, the addition of STDP based on the conventional drug treatment in the study group can significantly improve the coronary microvascular function compared with the control group (the key index is IMR).

### 2.3 Participant

We will enroll patients aged 18 to 80 years and the major clinical inclusion and exclusion criteria are listed in Table 2. In addition, for patients who meet clinical criteria, coronary angiography will be further performed. The inclusion criteria for the lesions are as follows: Coronary angiography shows diameter stenosis ≥50% in at least one of the three main coronary arteries with FFR ≥ 0.80. No need for revascularization (including balloon inflation, stent implantation, coronary artery bypass grafting, etc), IMR ≥ 25. At the same time, the following lesions need to be excluded:

- Thrombotic lesions;
- Coronary spasm without significant stenosis;
- Left main disease with a diameter stenosis rate of ≥50%;
- Type C-F dissection of coronary artery.

**Table 2:**
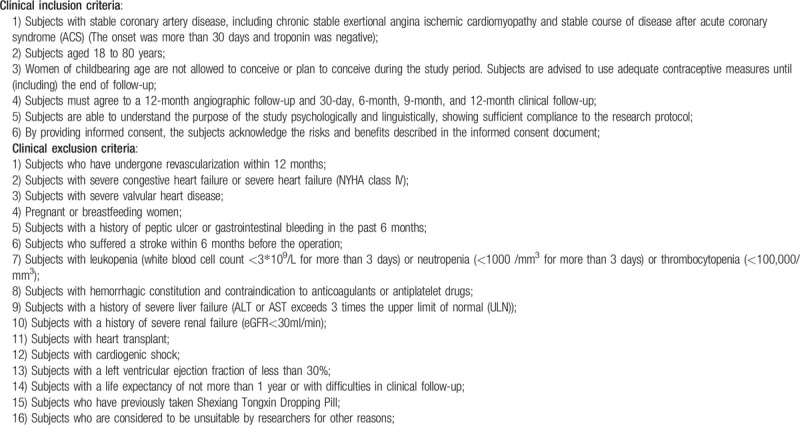
Clinical inclusion and exclusion criteria.

### 2.4 Recruitment

The study was carried out at Sir Run Run Show Hospital Affiliated to Zhejiang University School of Medicine. The patients interested in participating in the study will be evaluated by clinicians to determine their qualifications. Participants must give written informed consent which should be acquired after sufficient and detailed explanation to research situation (including confidentiality, risk and benefits, purpose of the study, etc).

### 2.5 Randomization

A computer-generated randomization schedule was prepared prior to recruitment by a statistician not otherwise involved in the trial, using stata 11 to implement a random number sequence according to simple randomization with a 1:1 allocation ratio. Group assignments were concealed in opaque sequentially numbered envelopes according to the randomization schedule. A print copy of the randomization schedule was stored in a sealed envelope under at research office. With a similar rate of recruitment over time into the two intervention groups, either randomization sequence would be expected to produce balanced intervention groups.

### 2.6 Intervention

The study group should be given routine medical treatment for SCAD (including but not limited to anti-platelet aggregation (such as aspirin, once daily, 0.1 g each time), lipid-regulating (such as atorvastatin, once daily, 20 mg each time) and other drugs), and on this basis, add STDP orally, three times a day, 70 mg each time; By contrast, the control group should be given routine medical treatment for SCAD (including but not limited to anti-platelet aggregation, lipid-regulating and other drugs).

### 2.7 Endpoints

#### 2.7.1 Primary and secondary endpoints

The primary endpoint is the change of Index of Microcirculatory Resistance (ΔIMR) at 12-month follow-up. The secondary endpoints are major adverse cardiovascular events (MACE, including cardiac death, myocardial infarction, ischemic stroke, unstable angina requiring hospitalization, coronary revascularization) at 12-month follow-up; the change in corrected TIMI frame count (ΔCTFC) after 1-year follow-up; the change in coronary flow reserve (ΔCFR) at 1-year follow-up.

#### 2.7.2 Safety endpoints

All parameters related to the participants’ safety including vital signs, physical examination, hematologic test, biochemical test, and judged cardiovascular events will be documented on the CRF at every visit. Any adverse events (AEs) that occur after taking test drug, regardless of the severity and whether they are related to the test drug, will be documented on the CRF and reported in a timely manner. If the AE is severe and related to STDP, the participant will be withdrawn from the study, and the follow-up and appropriate treatment will be provided to them.

### 2.8 Sample size

The sample size is calculated according to the main evaluation index ΔIMR at 12-month follow-up. Based on previous study, the level of ΔIMR is hypothesized to be 15.0 ± 6.0 and 10.0 ± 4.0 in study group and control group, respectively. When the calculation is performed using 90% power, and a 5% significance level (one-sided test), random distribution ratio is 1:1, the minimum number of members in each group is 25. Considering a drop-out rate of 20%, the needed sample size is approximately 32 participants for each group. Therefore, a total of 64 participants are needed for the present study.

### 2.9 Statistical analyses

#### 2.9.1 Descriptive analysis

Our descriptive statistics include that the count data is described by frequency and constituent ratio and the measurement data is described by mean, SDs, maximum value, minimum value, medians, 25^th^, and 75th percentiles.

#### 2.9.2 Baseline demographic analysis

Continuous correction χ^2^ test is used for comparison between count data groups. When the theoretical frequency of more than 25% of cells is <5, Fisher's exact probability method is used; comparison between groups of normally distributed measurement data uses group *t* test; for non-normally distributed measurement data, Wilcoxon rank sum test is used for comparison between groups.

#### 2.9.3 Efficacy analysis

Continuous correction χ^2^ test is used for comparison of MACE count treatment groups, paired *t* test is used for comparison of normal distribution measurement data groups; Wilcoxon sign rank is used for comparison of non-normal distribution measurement data groups (Wilcoxon Sign Rank) Test: The method of comparison between groups is the same as the baseline analysis.

#### 2.9.4 Safety evaluation

According to the study group and the control group, the number and proportion of abnormal cases before and after treatment are described respectively. AEs are described by the number and incidence of AEs, and the proportion is tested by continuous correction χ^2^ test or Fisher's exact probability method. At the same time, depict the specific manifestations, degree and relationship with the study drug of all AEs occurred in each group of cases in detail.

### 2.10 Data entry, monitor, and confidentiality

The case report form (CRF) will be finished by independent investigators in time, completely, correctly. After the completed CRF is reviewed by the clinical supervisor, it will be directly uploaded to the server through the Electronic Data Capture (EDC) system client and the database is password-protected. All records relating to the identity of the participant are kept confidential and the information will not be disclosed to the public beyond the limits permitted by relevant laws and/or regulations.

### 2.11 Ethics and dissemination

The protocol (version 2.0., April 8, 2020) is approved by the Ethics Committee of Sir Run Run Show Hospital Affiliated to Zhejiang University School of Medicine (No. 20200423–36). The study will be conducted in accordance with the principles of Good Clinical Practice (GCP) and the Declaration of Helsinki. Written informed consent will be obtained from all patients, who will be assigned a unique identifier; Study findings will be disseminated through presentations at national and international conferences and publications in peer-reviewed journals.

## 3 Discussion

CMVD is a research hot spot in the field of cardiovascular disease. Previous studies have shown that the incidence of CMVD is 51% in men and 54% in women.^[8]^ However, in China, only 6.3% of patients with CMVD get correct diagnosis and treatment. Among them, for some patients with SCAD and coronary angiography showing epicardial CAD but with normal FFR, it is easier to ignore the detection and timely treatment of CMVD, which may cause serious consequences.

Currently, there are no effective therapies specifically targeting CMVD. Optimized medical therapy of myocardial protection while controlling risk factors is an important goal of treatment. Traditional anti-ischemic drugs including beta-blockers and short-acting nitrates, should be considered to be the first choice of therapy in CMVD.^[9]^ Calcium antagonists and long-acting nitrates may be helpful when used in addition to beta-blockers in the case of insufficient control of symptoms. Calcium antagonist, however, can be first-line therapy in patients with angina pectoris with microvascular spasm.^[9]^ Angiotensin-converting enzyme inhibitor (ACEI) may help reverse vascular hypertrophy and improve vascular compliance via improve endothelial dysfunction and vasoreactivity.^[5]^ New anti-ischemic drugs such as ranolazine or ivabradine have been proven effective in some patients with microvascular angina.^[10–12]^ ESC guidelines recommend the use of secondary prevention with aspirin and statin therapy. Nevertheless, the efficacy of above-mentioned drug is not satisfactory for some patients with CMVD, far less than that of angina pectoris in patients with obstructive CAD.

STDP is a formulate traditional Chinese Medicine widely used to treating clinical cardiovascular disease, especially in stable angina pectoris in China and South Asia.^[13–16]^ It is a complex herb formula, ultra performance liquid chromatography coupled with triple-quadrupole tandem mass spectrometry (UPLC QqQ-MS/MS) Quantitative Analysis revealed 13 major chemical constituents in STDP,^[17]^ which has the functions of dilating blood vessels, anti-inflammatory and reducing endothelial damage. Previous studies have revealed that STDP has a significant improvement on myocardial ischemia. In the rat model of acute myocardial ischemia induced by Pituitrin, the application of STDP can prevent ST segment elevation, reduce whole blood viscosity, and serum CK-MB and LDH levels were lower, result in down-regulation of Bax expression and up-regulation of Bcl-2 expression in myocardial tissue.^[18]^ At the same time, a study explored the probable mechanism of STDP to protect myocardial ischemia, which discovered that STDP protected against the ISO-induced myocardial ischemic injury via an ERK1/2 signaling pathway, thereby providing a mechanism to support clinical applications of STDP as treatment for ischemic heart disease.^[19]^ In addition, STDP can also alleviate atherosclerotic lesions, which has been confirmed in ApoE-deficient mouse models.^[20]^ Related pharmacological studies have proposed that STDP can protect endothelial cells from atherosclerotic lesions by decreasing levels of endothelin-1 (ET-1), C reactive protein (CRP), tumor necrosis factor-α (TNF-α) and increasing levels of nitrogen oxide (NO) in the blood,^[21]^ indicating its potential effect on the pathophysiology of atherosclerosis, including lipid regulation, fibrosis, inflammation and oxidative stress.^[22]^

As mentioned above, the effect of STDP on improving the coronary microvascular function is worthy of expectation. Our study will be the first randomized controlled study to investigate the effect of STDP in SCAD patients with normal FFR and CMVD. By assessing clinical efficacy as well as safety of CMVD, the data obtained may guide STDP use in CMVD.

## Acknowledgments

The authors thank the Sir Run Run Show Hospital Affiliated to Zhejiang University School of Medicine for providing a platform and instruments for this study.

## Author contributions

Contributors: FYQ conceived and designed the study. YLL was responsible for ET-1, NO, TNF-α detection and drafted the protocol presented. JHZ, CYJ, and GSF were responsible for coronary angiography and FFR, CFR, IMR test. XPC provided statistical support. YBZ was in charge of clinical follow-up. All authors critically reviewed and approved the final version of the manuscript.

## Abbreviations

Abbreviations: AEs = adverse events, CAD = coronary artery disease, CFR = coronary flow reserve, CMR = cardiovascular magnetic resonance, CMVD = coronary microvascular disease, CRF = case report form, CTFC = corrected TIMI frame count, FFR = fractional flow reserve, IMR = index of microcirculatory resistance, MACE = Major Adverse Cardiovascular Events, SAEs = Severe Adverse Events, SCAD = stable coronary artery disease, STDP = Shexiang Tongxin dropping pill, TIMI = Thrombolysis in Myocardial Infarction.
